# Editorial: Rising stars in neuropsychology 2021

**DOI:** 10.3389/fpsyg.2024.1382852

**Published:** 2024-02-26

**Authors:** Micaela Mitolo, Daniela Smirni, Matteo De Marco, Nicola Canessa, Sara Palermo

**Affiliations:** ^1^Department of Medicine and Surgery, University of Parma, Parma, Italy; ^2^Functional and Molecular Neuroimaging Unit, Istituto di Ricovero e Cura a Carattere Scientifico (IRCCS) Istituto delle Scienze Neurologiche di Bologna, Bologna, Italy; ^3^Psychological, Pedagogical, Physical Exercise and Training Sciences, University of Palermo, Palermo, Italy; ^4^Division of Psychology, Brunel University London, Uxbridge, United Kingdom; ^5^University School for Advanced Studies, University Institute of Higher Studies in Pavia, Pavia, Italy; ^6^Department of Psychology, University of Turin, Turin, Italy; ^7^Neuroradiology Unit, Diagnostic and Technology Department, Fondazione Istituto di Ricovero e Cura a Carattere Scientifico (IRCCS) Istituto Neurologico Carlo Besta, Milan, Italy

**Keywords:** neuropsychology, Adult Attention-Deficit/Hyperactivity Disorder (ADHD), Primary Progressive Aphasia (PPA), linguistic, executive (dys)functions

In the kaleidoscope of neuropsychological research, the *Rising stars in neuropsychology 2021* Research Topic converges on pivotal studies exploring Adult Attention-Deficit/Hyperactivity Disorder (ADHD), the role of predictive processing in linguistics, and the variants of Primary Progressive Aphasia (PPA). In this editorial, we delve into these studies while weaving connections with executive functioning, neurodegenerative diseases, and the nuanced framework of neurocognitive models.

Ceroni et al. investigated how adult ADHD transcends symptomatology, delving into the neurocognitive intricacies. This study invites a deeper exploration of how executive functions, often implicated in ADHD, align with the broader neurocognitive model of this disorder. Incorporating insights from neurocognitive models could offer a holistic understanding of the neuropsychological profile of ADHD, unraveling the underlying cognitive processes that contribute to attention and executive function deficits observed in this population.

Grisoni examined how predictive processing in linguistics holds implications for neurocognitive models. The anticipation and interpretation of linguistic input likely involve the orchestrated interplay of executive functions within a broader cognitive framework. Linking these findings to established neurocognitive models can offer a unified perspective, elucidating how predictive processing aligns with executive functioning and contributes to the broader cognitive architecture.

Lukic et al. offered a unique lens into the neurodegenerative spectrum of language disorders. Integrating neurocognitive models into the discussion allows for a more comprehensive understanding of how executive functions become entwined with linguistic processing and neurodegeneration. Unraveling the distinct error patterns in PPA variants within the context of neurocognitive models may elucidate the specific cognitive components affected and contribute to a more refined diagnostic framework.

The Rising Stars Research Topic, when viewed through the lens of comprehensive neurocognitive models, propels us toward a deeper understanding of the interconnectedness between executive functions, linguistic processing, and neurological conditions. This synthesis beckons researchers to embark on a collaborative journey, fusing insights from diverse domains to construct a more unified and nuanced framework for comprehending the intricacies of the human brain and its pathologies. Within this paradigm, recent works contribute exploring the intricate relationship between executive and metacognitive dysfunction in neurological disorders, illuminating the challenges faced by individuals grappling with these conditions (Amanzio et al., 2017, 2020). Another sheds light on the potential role of metacognitive dysfunction and mood changes as correlates of pre-frailty in neurocognitive disorders, adding layers to our understanding of the cognitive aspects intertwined with neurological conditions (Morese et al., 2018).

Collectively, these insights from recent literature amplify the call for exploration, resonating with the promise of unveiling the mysteries at the intersection of cognition, pathology, and the evolving landscape of neuropsychology.

## Author contributions

MM: Writing—review & editing. DS: Writing—review & editing. MD: Writing—review & editing. NC: Writing—review & editing. SP: Conceptualization, Writing—original draft.
